# A vision of the future for BMC Medicine: serving science, medicine and authors

**DOI:** 10.1186/1741-7015-7-55

**Published:** 2009-10-07

**Authors:** Robin L Cassady-Cain, Joanne M Appleford, Jigisha Patel, Mick Aulakh, Melissa L Norton

**Affiliations:** 1 BMC Medicine Editorial team, BioMed Central Ltd, 236 Gray's Inn Road, London WC1X 8HL, UK

## Abstract

In June 2009, *BMC Medicine *received its first official impact factor of 3.28 from Thomson Reuters. In recognition of this landmark event, the *BMC Medicine *editorial team present and discuss the vision and aims of the journal.

## Editorial

*BMC Medicine *recently celebrated its 5^th ^anniversary of publishing high quality research with an editorial reflecting on our progress and on developments for the journal in 2009[1]. Now another highly anticipated milestone has been achieved with the announcement of the journal's first "official" impact factor of 3.28 by Thomson Reuters.

While one can discuss the pros and cons of the impact factor system, and others have done so tirelessly [[2-4] for example], it cannot be denied that the receipt of an impact factor is a major event in the life of a young journal. As such, this is an opportune moment to present our vision and aims for the future of *BMC Medicine*.

In this era of translational medicine, clinicians must understand the latest relevant information that may influence the practice of medicine. Whether one is a practicing physician, a clinical researcher, or a member of the lay public, understanding how specific scientific research will affect clinical practice may be difficult, especially when that research comes from an area outside of one's own personal experience and expertise. We expect that *BMC Medicine *will play an increasingly important role in facilitating the understanding of biomedical discovery and in appreciating the connection between these discoveries and clinical practice. This understanding and appreciation will guide future research in all fields of investigation that impact on the management of human disease.

The journal will accomplish this through the publication of high quality research, supported by interpretation of this work in accompanying reviews, minireviews and commentaries to outline the wider implications and potential future directions of the research. We are committed to publishing high quality studies that fall into one of three general categories: clinical investigations with clear and immediate ramifications for medical practice, clinical research that does not have immediate ramifications for medical practice, but that will inform the direction of future research, or basic biomedical work with a strong potential to influence clinical research.

We see a need for increased collaboration between basic and clinical researchers, and we believe that *BMC Medicine *can facilitate this process. In order to achieve this, we will publish our basic biomedical research articles with accompanying pieces highlighting the importance of this research to a wider audience and outlining how the research might inform clinical research or practice in the future. An example of this is the commentary by Rejko Kruger[5], which explains how the results of Jeanne Latourelle and colleagues' research on genetic mutations in Parkinson's Disease (PD) [6] increase our understanding of gene penetrance in PD and comments on how those results relate to current ideas about genetic counseling for PD.

Further, the addition of reviews and minireviews summarising important developments in current key fields will increase the accessibility of research and educate non-experts regarding how current research affects clinical practice or disease management, For example, the recent review by Brian Coburn and colleagues discusses the use of mathematical modeling to predict the disease course of swine flu as well as outlining the important parameters for pandemic preparedness[7].

No vision of the future of *BMC Medicine *can be complete without considering our authors. As editors, our service to *BMC Medicine's *authors is as important as our service to the medical community. Therefore we will continue to uphold the three principles recently expounded by Raff and colleagues [8]: that the peer review process should be fast, friendly and fair.

Research needs to be published in such a way as to ensure that the importance, timeliness and potential of the findings are clear to the entire medical community. The editors of *BMC Medicine *are committed to the rapid dissemination of research throughout both the biomedical and clinical communities worldwide, while continuing to provide an excellent and efficient service to our authors. We look forward to working with you, and for you, in the times to come.

## Pre-publication history

The pre-publication history for this paper can be accessed here:


